# Reliability and Validity of the Double Inclinometer Method for Assessing Thoracolumbar Joint Position Sense and Range of Movement in Patients with a Recent History of Low Back Pain

**DOI:** 10.3390/healthcare11010105

**Published:** 2022-12-29

**Authors:** Zacharias Dimitriadis, Ioannis Parintas, Georgios Karamitanis, Kiven Abdelmesseh, George A. Koumantakis, Alexandros Kastrinis

**Affiliations:** 1 Health and Quality of Life Assessment Research Lab, Physiotherapy Department, University of Thessaly, 35132 Lamia, Greece; 2 Laboratory of Advanced Physiotherapy, Physiotherapy Department, School of Health & Care Sciences, University of West Attica (UNIWA), 12243 Athens, Greece

**Keywords:** dual inclinometer, dual inclinometry, lumbar, kinesthesia, proprioception, range of motion, repeatability, reproducibility, Schober’s test, spinal pain

## Abstract

The study was aimed at examining the reliability of the Double Inclinometer (DI) method for the assessment of thoracolumbar Range of Movement (ROM) and Joint Position Sense (JPS) in patients with a recent history of Low Back Pain (LBP). Twenty patients with a history of LBP in the last three months participated. The thoracolumbar ROM and JPS were examined from a standing position by using both the DI and the tape measure method. The DI method was found to have moderate to good intra-rater (ICC = 0.68–0.79, SEM = 2.20–2.77°, SDD = 6.09–7.67°), moderate inter-rater (ICC = 0.59–0.62, SEM = 2.96–3.35°, SDD = 8.19–9.27°) and poor test-retest reliability (ICC = 0.13–0.17, SEM = 3.98–4.32°, SDD = 11.02–11.96°) for the assessment of thoracolumbar JPS. For the assessment of thoracolumbar ROM, the DI method was found to have good to excellent intra-rater (ICC = 0.88–0.94, SEM = 4.25–6.20°, SDD = 11.77–17.17°), excellent inter-rater (ICC = 0.90–0.91, SEM = 7.26–7.74°, SDD = 20.11–21.43°) and excellent test-retest reliability (ICC = 0.91–0.93, SEM = 6.03–6.87°, SDD = 16.70–19.02°). The concurrent validity of the DI method with the tape measure method was found to be very weak for the assessment of thoracolumbar JPS (r = 0.02, *p* = 0.93) and strong for the assessment of thoracolumbar ROM (r = 0.66, *p* = 0.001). The DI method seems to be a very reliable method for the assessment of thoracolumbar ROM in individuals with a recent history of LBP.

## 1. Introduction

Low Back Pain (LBP) is an extremely common musculoskeletal complaint and is the leading cause of work absence and activity limitation in a great part of the world. The 1-year incidence of a first-ever episode of LBP is 6.3–15.4% and the 1-year incidence of any episode of LBP is 1.5–36%. Estimates of 1-year recurrence range from 24% to 80%. The point prevalence has been estimated between 1% and 58.1% and the 1-year prevalence between 0.8% and 82.5% [1].

Patients with LBP may also have a number of clinical manifestations including reduced muscle strength of the trunk and the lower extremities [2], increased postural sway [3], impaired motor control [4], changes in posture [5], psychological compromise [6] as well as affected spinal Range of Movement (ROM) [3] and changes in sensorimotor control [7]. The impaired proprioception of patients with spinal pain may affect the informative input to the central nervous system and lead to changes in the control of movement, the recruitment strategy and the accuracy of motor commands influencing the posture, therefore, contributing to spinal pain [8,9]. Therefore, the assessment of lumbar and thoracolumbar proprioception seems to be very important for the optimal management of patients with LBP.

The assessment of lumbar and thoracolumbar proprioception has been performed with a number of measurement procedures and equipment [10]. Joint Position Sense (JPS) is the aspect of proprioception that is most frequently examined in patients with LBP and it is usually examined with the active repositioning of the spine in a predetermined target position [10]. By using this method, the deviation of lumbar or thoracolumbar repositioning from the predetermined position represents the magnitude of the deficiency in JPS [11].

The assessment of JPS necessitates tools that are able to provide recordings of the corresponding ROM. Inclinometers and tape measures are tools that have been frequently used, especially in clinical practice, to assess lumbar or thoracolumbar ROM [12] due to their affordable, easily applicable and non-invasive nature. Digital inclinometers may offer more objective recordings, as the numbers of the conventional inclinometers may be more difficult to read and might be more dependent on the examiner’s judgment. Furthermore, digital inclinometers may offer more opportunities to the users such as calibration at an angle of interest, the holding of the measurement, lights and other technological feats. Although spinal JPS can be assessed with the use of a single inclinometer [13], the use of the double inclinometer method [14] seems to be preferable. The double inclinometer method is a popular method for the assessment of lumbar ROM [15], but it has been also occasionally used for the assessment of lumbar proprioception [14]. This method seems to be better than the single inclinometer method as the second inclinometer can be used as a reference in order to exclude the influence from any other unwanted movement of the pelvis and lower limbs.

Tape measures can be used to assess the changes in the spinal curvature by measuring either the distance of an anatomical landmark, which is not necessarily related to the spine, from a predetermined environmental landmark [16] or the change in distance between specific anatomical landmarks of the spine [17]. The well-known Schober’s test is a frequently used diagnostic procedure for the measurement of lumbar flexion by following the latter concept [18].

Although the double inclinometer and the tape measure method have been used in clinical practice and research for the assessment of lumbar/thoracolumbar ROM and JPS in healthy people and patients with LBP, the evidence about their reliability and validity is more than limited. More specifically, according to our knowledge, the evidence about the reliability of the double inclinometer method for the examination of lumbar/thoracolumbar JPS in patients with LBP seems to be non-existent. Therefore, the aims of the study were: (a) to examine the intra-rater, inter-rater and test-retest reliability of the double inclinometer method for the assessment of thoracolumbar JPS and ROM and (b) to examine the concurrent validity of the double inclinometer method with the tape measure method for the assessment of thoracolumbar JPS and ROM.

## 2. Materials and Methods

### 2.1. Sample

Twenty patients with a recent lumbar pain history participated in the study. The sample was recruited with a convenience sampling method. Participants were included if they (a) had a history of LBP in the last three months, (b) were between 18 and 65 years old and (c) had sufficient cognitive ability and knowledge of the Greek language to complete the questionnaires and sign an informed consent. Participants with serious spinal or other pathologies, spinal surgeries, malignancies, systemic diseases and/or any other neurological, respiratory, cardiovascular and orthopedic disorder that could influence movement and the perception of movement were excluded from the study. The assessments were performed at an appropriately configured lab of the Physiotherapy Department, University of Thessaly, Lamia, Greece. Before their participation, the volunteers had to read an information sheet and provide their written informed consent. The study was conducted according to the guidelines of the Declaration of Helsinki and was approved by the Deontology Committee of the Physiotherapy Department, University of Thessaly, Lamia, Greece (2378ΣΕ1/22-02-2022).

### 2.2. Equipment and Materials

**Figure 1:**
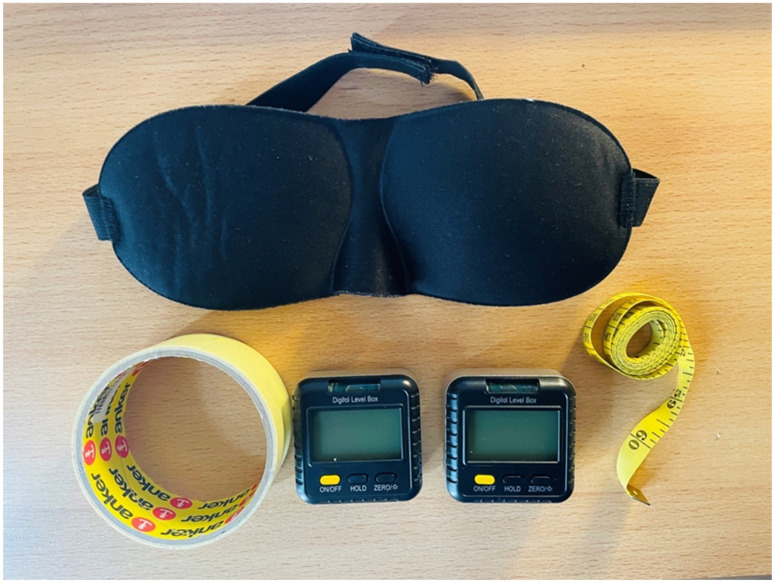
The basic equipment of the study. The basic equipment was a blindfold (top), adhesive (bottom left), two inclinometers (bottom middle) and a tape measure (bottom right).

The basic equipment of the study is presented in Figure 1. For the assessment of thoracolumbar proprioception and ROM, two digital inclinometers were used (Digital level box, eSynic, UK). The inclinometers provide a recording in degrees (°). Their front and rear panels are made of plastic and the frame is made of aluminum alloy. The dimensions of each inclinometer were 6.6 × 6.5 × 3.4 cm and the weight 70 g. It has been described that they have a measuring range of 4 × 90°, accuracy ±0.2° (±0.1° at 0° and 90°), and a resolution of 0.05°. A conventional tape measure was also used for the same measurements (Profi-Prym, Stolberg, Germany). A blindfold was used for negating the vision of the participants during the performance of the tests (Sleepo, Serres, Greece). A double-sided adhesive was used for the fixation of the inclinometers at the predefined anatomical landmarks.

Pain intensity was recorded with the Numeric Pain Rating Scale (NPRS). The NPRS used was a segmented scale—from 0 (no pain at all) to 10 (worst possible pain)—in which each participant was asked to select the number that more appropriately described his/her pain intensity [19].

The participants’ disability was assessed with the Greek version of the Oswestry Disability Index (ODI) [20]. The ODI was initially published in English [21]. It includes 10 items with questions regarding pain intensity, personal care, lifting, walking, sitting, standing, sleeping, sex life, social life and traveling. Each item has six potential responses and their score may range from 0 to 5. The total score of the instrument may range from 0 (no disability) to 50 (maximal disability). The score can be also multiplied by 2 in order to be expressed as a percentage. In this case, the scores of the instrument can be interpreted as follows: 0–20% minimal disability, 21–40% moderate disability, 41–60% severe disability, 61–80% crippled, 81–100% complete disability [21,22]. The instrument has been found to have satisfactory psychometric properties for Greek patients with LBP [20].

### 2.3. Procedure

The participants were initially asked to complete demographics, general health and pain condition questionnaires as well as the NPRS and ODI. Then, the thoracolumbar JPS and ROM were assessed.

**Figure 2:**
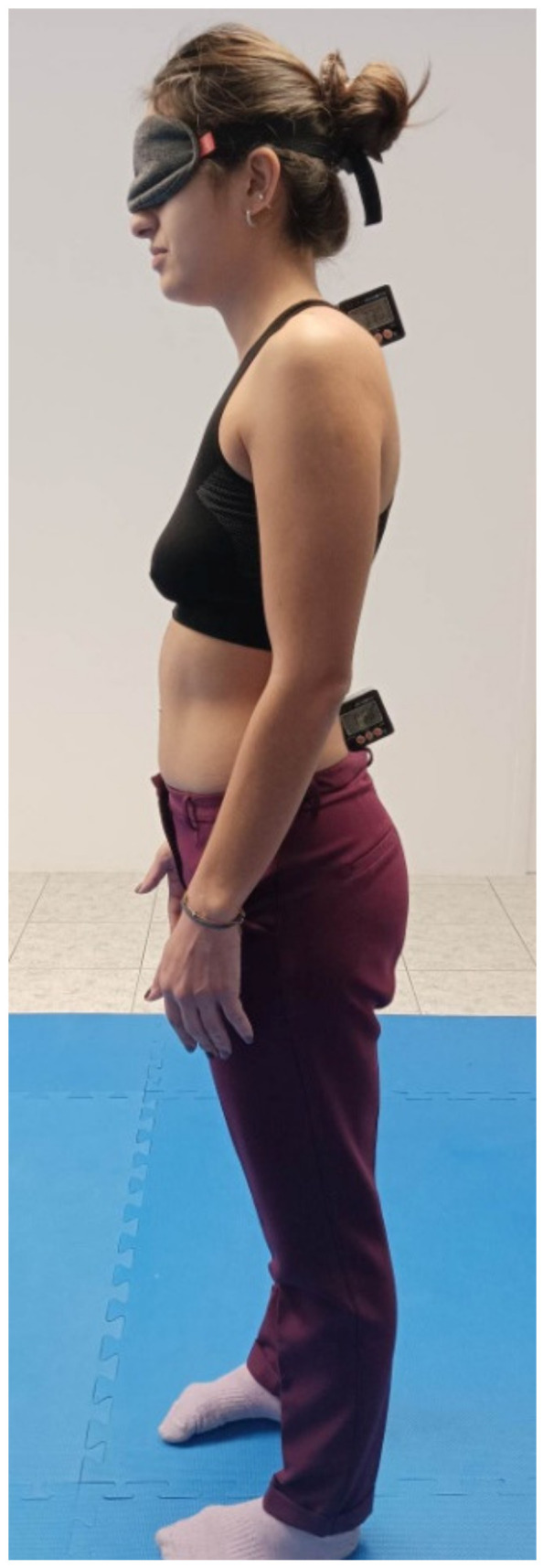
Initial position and placement of the inclinometers.

Initially, the participants were asked to lie down in a prone position on a plinth. From this position, the first rater (Rater A) palpated and marked the first sacral (S1) and the seventh cervical (C7) vertebra. Then, the participants were asked to stand up and the two digital inclinometers were fixed to these anatomical landmarks with the use of double-sided adhesive tapes (Figure 2). The cervical inclinometer would help for the recording of the spinal movement, whereas the sacral inclinometer would serve as a reference inclinometer for excluding movement from any other body parts (e.g., from the hip).

**Figure 3:**
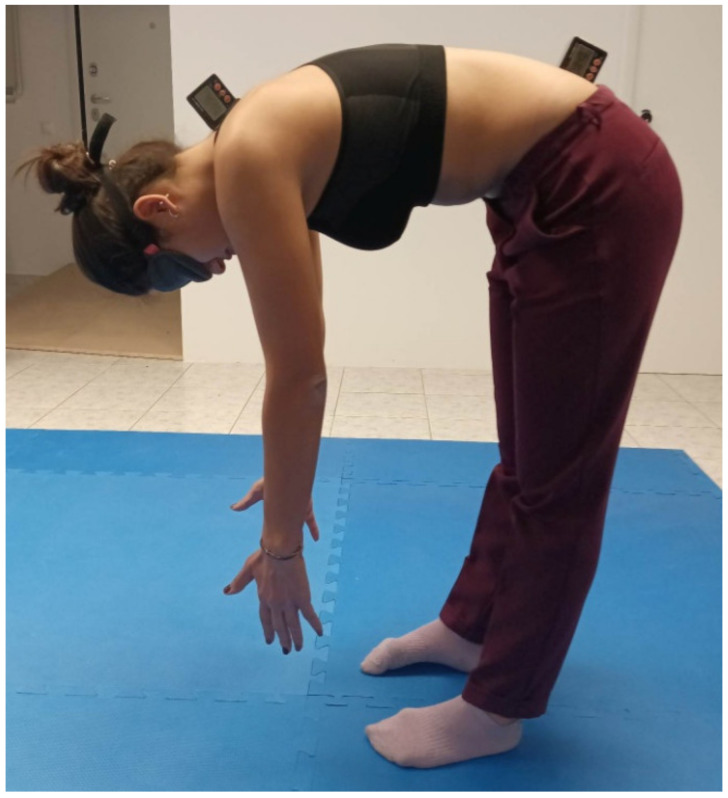
Measurement of thoracolumbar range of movement by using the double inclinometer method.

The patients were standing with their feet apart from each other at a distance equal to the width of the shoulders. From this standing natural position (starting position), the participants were asked to flex their spine as much as possible by moving toward the ground and the thoracolumbar ROM was determined from the difference in the recordings between the two inclinometers (Figure 3). This was repeated three times.

**Figure 4:**
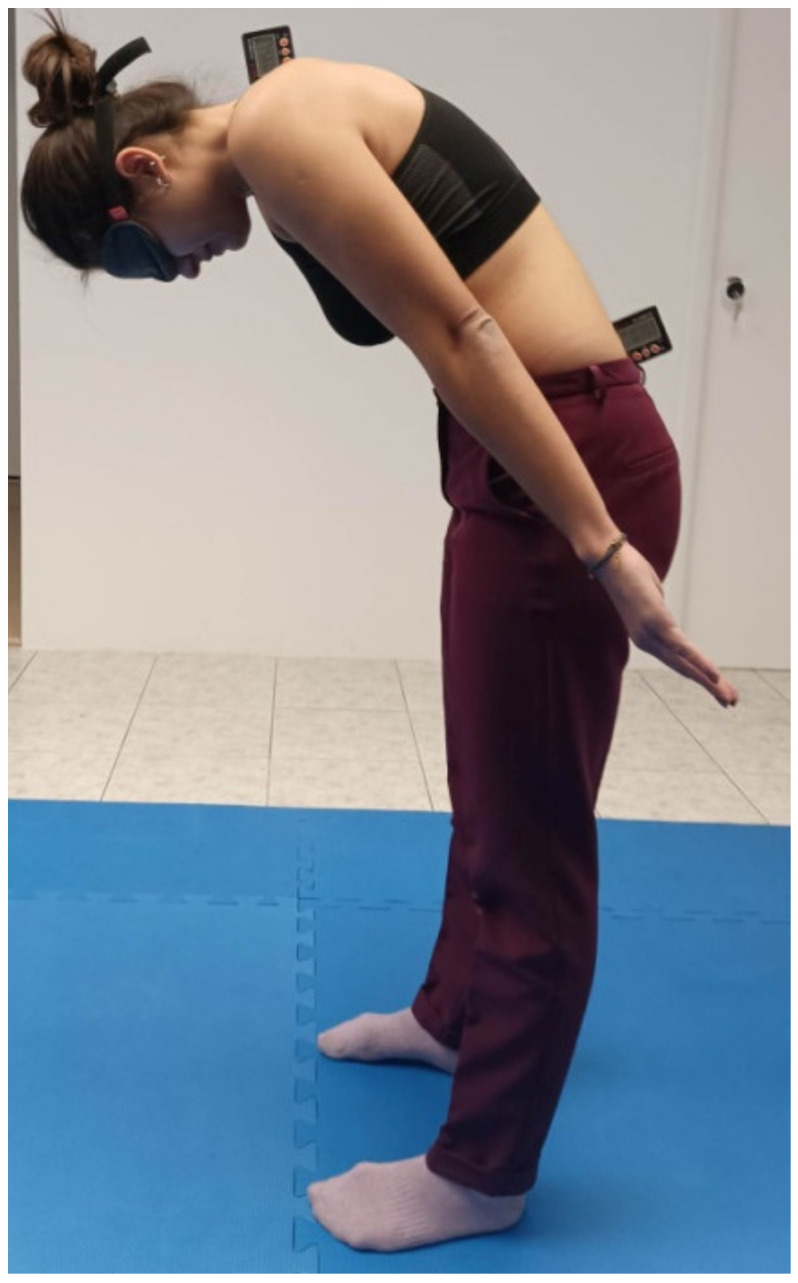
Measurement of thoracolumbar joint position sense by using the double inclinometer method.

Then, a blindfold was applied to the participants and they were actively and slowly positioned in 40° of thoracolumbar flexion (target position). The participants were asked to keep this position for about 5 s to memorize it and then, they returned to the starting natural position. Then, the participants were asked to perform thoracolumbar flexion and find the target position (Figure 4). When they confirmed that they had assumed the predetermined position, the inclinometers were stabilized and their recordings helped to determine the thoracolumbar ROM that was performed by the participants. The absolute difference (absolute error) between the target position and the position achieved by the participants was used as the index of JPS. Five trials of thoracolumbar repositioning were performed. After a break of 15 min, the procedure was repeated by a second rater (Rater B). Then, 15 min after the completion of the measurements by the Rater B, Rater A repeated the measurements in exactly the same way.

A third rater (Rater C) was also used to assess the thoracolumbar JPS and ROM with the tape measure method, in a way quite similar to Schober’s test [18]. Initially, from the same natural standing position as before, a tape measure was used to measure the distance between the C7 and S1. Then, the participants were asked to perform a maximal thoracolumbar flexion and the tape measure was used to record the new distance between C7 and S1. The difference between the S1-C7 distance at the natural standing position and the S1-C7 distance and the position of maximal thoracolumbar flexion was the index of thoracolumbar ROM. Three trials of this test were performed.

Then, a Rater C assessed the thoracolumbar JPS with the same tape measure. For this test, a blindfold was applied to the participants and the tape measure was applied to measure the distance between S1 and C7. Then, the participants were slowly and actively positioned in thoracolumbar flexion so that the recording of the tape measure was increased by 5 cm (target position). After the recording of the S1-C7 distance (target position), the tape measure was removed and the participants were asked to keep this position for about 5 s to memorize it. Then, they returned to the starting natural position. From this position, the participants were asked to perform thoracolumbar flexion and find the target position. When they confirmed that they had assumed the predetermined position, the tape measure was reapplied to record the distance between the S1 and C7. The absolute difference (absolute error) between the target position and the position achieved by the participants was used as the index of JPS. Five trials of thoracolumbar repositioning were performed.

The three raters were blind to the measurements of each other.

### 2.4. Data Analysis

The normality of data was examined with Kolmogorov–Smirnov tests. Intra-rater reliability of the double inclinometer method for the assessment of both thoracolumbar JPS and ROM was examined with the Intraclass Correlation Coefficient type 2,1 (ICC_2.1_). Inter-rater and test-retest reliability were examined with the ICC_2,k_. ICC values of 0–0.5, 0.5–0.75, 0.75–0.9, and 0.9–1 were considered indicative of poor, moderate, good and excellent reliability, respectively [23].

Standard Error of Measurement (SEM) was calculated as the square root of the within groups mean square. Smallest Detectable Difference (SDD) was calculated by the equation SEM ∗ 1.96 ∗ √2. SEM and SDD interpretation seem to be quite arbitrary [24]. However, some suggestions have been provided for their interpretation. Therefore, for this study, SEM values of <15% of the grand mean were considered satisfactory [25]. Furthermore, SDD values of <30% were considered satisfactory and of <10% were considered excellent [24].

The concurrent validity of the double inclinometer with the tape measure method for the assessment of thoracolumbar JPS and ROM was examined by using Pearson (r) correlation coefficients. These associations could be interpreted as very weak (0–0.19), weak (0.20–0.39), moderate (0.40–0.59), strong (0.60–0.79), or very strong (0.80–1.00) [26].

The significance level was set at *p* = 0.05. The IBM SPSS for Windows, version 22.0 (IBM Corp., Armonk, NY, USA) was used for all statistical analyses.

## 3. Results

The sample of the study could be described as a mixed sample of young men and women, of moderate height and weight and of moderate usual pain intensity and minimal disability. The demographics of patients with LBP are presented in Table 1.

For the assessment of thoracolumbar JPS, the intra-rater reliability of the double inclinometer method was generally found to be moderate to good (ICC = 0.68–0.79, SEM = 2.20–2.77°, SDD = 6.09–7.67°). Inter-rater reliability was generally found to be moderate (ICC = 0.59–0.62, SEM = 2.96–3.35°, SDD = 8.19–9.27°). Test-retest reliability was generally found to be poor (ICC = 0.13–0.17, SEM = 3.98–4.32°, SDD = 11.02–11.96°). The findings about the reliability of the double inclinometer method for the assessment of thoracolumbar JPS are presented in detail in Table 2, Table 3 and Table 4.

For the assessment of thoracolumbar ROM, the intra-rater reliability of the double inclinometer method was generally found to be good to excellent (ICC = 0.88–0.94, SEM = 4.25–6.20°, SDD = 11.77–17.17°). Inter-rater reliability was generally found to be excellent (ICC = 0.90–0.91, SEM = 7.26–7.74°, SDD = 20.11–21.43°). Test-retest reliability was generally found to be excellent (ICC = 0.91–0.93, SEM = 6.03–6.87°, SDD = 16.70–19.02°). The findings about the reliability of the double inclinometer method for the assessment of thoracolumbar ROM are presented in detail in Table 5, Table 6 and Table 7.

The concurrent validity of the double inclinometer method with the tape measure method was found to be very weak for the assessment of thoracolumbar JPS (r = 0.02, *p* = 0.93) and strong for the assessment of thoracolumbar ROM (r = 0.66, *p* = 0.001).

## 4. Discussion

The double inclinometer method was found to have moderate to good intra-rater reliability, moderate inter-rater reliability and poor test-retest reliability for the assessment of thoracolumbar JPS. Interestingly, inter-rater reliability was found to be better than the test-retest reliability; the opposite was expected due to the fact that different raters conducted the assessment, including palpation and placement of the markers. This finding might be attributed to testing effects as the measurements for the test-retest reliability were performed last and the patients might have felt boredom, tiredness, or changes in their pain condition. The SDD and the SEM values were found to be rather unacceptable since their values were more than 30% of their corresponding grand means. Interestingly, although the double inclinometer method has been used to assess lumbar proprioception [14,27], according to our knowledge, there is no other evidence to compare the results of the current study. However, based on the current findings, it seems that the reproducibility of the double inclinometer method may be acceptable, but the large error of measurement and the poor test-retest reliability suggests that further investigation and refinement of the method are needed until more acceptable values are obtained.

The concurrent validity of the double inclinometer with the tape measure method for the assessment of thoracolumbar JPS was found to be very weak. This is not necessarily a negative finding for the double inclinometer method as the clinometric properties of the tape measure for the assessment of thoracolumbar proprioception are also not established. However, although the non-satisfactory reliability indices may have contributed to the very low association between the two methods, the hypothesis that the two methods may measure different concepts or different dimensions of the same concept cannot be ignored.

In terms of the assessment of thoracolumbar ROM, the double inclinometer method was found to have good to excellent intra-rater reliability and excellent inter-rater and test-retest reliability. The current findings about the intra-rater reliability are in agreement with the findings of another study [15] (ICC = 0.78–0.91) in a mixed sample of patients with LBP and healthy individuals. On the contrary, the inter-rater reliability of the other study [15] (ICC = 0.60–0.66) was worse than the one found in the current study. However, such differences may be explained by the fact that the other researchers [15] examined the lumbar JPS rather than the thoracolumbar JPS which was examined in the current study. The findings of the current study are also better than the findings of another older study [28], which examined the reliability of the double inclinometer method with non-digital inclinometers for the assessment of lumbar ROM in patients with LBP (inter-rater: ICC = 0.60, test-retest: r = 0.13–0.87).

The SEM of the double inclinometer method for assessing the thoracolumbar ROM was completely satisfactory as it was never more than 15% of the grand mean. SDD values were also satisfactory for the intra-rater and test-retest reliability as they always remained below 30% of the grand mean, and only for the inter-rater reliability they were slightly more (31–33% of the grand mean). Unfortunately, none of the previously discussed studies [15,28] provide SEM and SDD values for a direct comparison with the current findings.

The concurrent validity of the double inclinometer with the tape measure method for the assessment of thoracolumbar ROM was found to be significantly strong. The use of a tape measure for the assessment of lumbar ROM has known reliability [12] and can provide support to the validity of the double inclinometer method for the assessment of lumbar ROM. In a previously performed study [29], the association of the two methods for the assessment of lumbar ROM was found to be weak (r = 0.01–0.32). However, this difference may be partially attributed to the different types of inclinometers, the different tape measure methods and the examination of different spinal regions in these two studies. Nevertheless, the current findings are quite promising for the use of the double inclinometer method for the assessment of thoracolumbar ROM.

The findings of the study are rich in clinical implications. The thoracolumbar spine is a region that has been found to have impaired proprioception and reduced ROM in patients with LBP [7,30,31]. The study provides a reliable method for assessing thoracolumbar ROM with a measurement tool that is cost-efficient, time-efficient and easily applicable. This is especially important for clinicians who do not have access to heavy and expensive instrumentation. Furthermore, the small SEM and satisfactory SDD values give clinicians the opportunity to conduct more accurate judgments on the effectiveness of their therapeutic approaches. However, the appropriateness of the double inclinometer method for assessing thoracolumbar proprioception in clinical practice is questionable. This is mainly due to the findings on its absolute reliability, as its relative reliability seems to be occasionally acceptable. A potential increase in the number of trials would help to decrease the random error of the measurements [32], but this is something that necessitates further investigation before suggesting confidently its use in clinical practice.

Despite the important clinical implications, the study has some limitations that should be thoughtfully considered before the absolute adoption of the findings. The sample size of the study was not calculated based on statistics and may seem to be relatively small. This might partially explain the wide confidence intervals of the ICCs. The double inclinometer method was compared with the tape measure method for establishing their concurrent validity. Although the examination of this association provides important information, the method was compared with another method that has not been examined for its reliability for the measurement of thoracolumbar JPS. However, the limited evidence for the reliability of other easily applicable clinical methods and the lack of an actual “gold standard” for assessing thoracolumbar JPS [7], render the selection of an absolutely appropriate reference assessment tool difficult. Another limitation is that the retest session was selected to be performed the same day with a very narrow interval between the test and retest session. This selection might be responsible for the non-satisfactory test-retest reliability due to potential testing effects. However, this interval was mainly selected in order to reduce the threat of drop-outs as well as to have better control of the patients’ activities between the sessions.

The study only included the assessment of thoracolumbar flexion. This movement was selected for examination due to its greater range of movement and its clinical relevance with low back pain. The ROM and JPS in other directions could also be examined, but it was avoided in order to reduce the testing effects to the minimum. Another parameter that should be considered is that the examined procedure did not take into account movements in other planes. However, this was not the intention of the study, movements in other planes cannot be avoided and their recording necessitates much more complex equipment and procedures.

Future studies should examine the validity of the double inclinometer method by comparing its recordings with the ones that are produced by more heavy and accurate instrumentation. The examination of the clinometric properties of the tape measure method for the assessment of spinal JPS should also comprise a field of future research as it is a very time-effective, cost-effective and easily applicable procedure with relatively no evidence about its ability to assess spinal proprioception. These methods should be also investigated in clinical populations other than patients with LBP. For example, the investigation of their clinometric properties in scoliosis could be especially meaningful and important. The examination of such research questions with the parallel elimination of the methodological weaknesses observed in the current study could significantly improve our knowledge of proprioception assessment, leading to better therapeutic outcomes.

## 5. Conclusions

The double inclinometer method is a very reliable procedure for the assessment of thoracolumbar ROM in patients with a recent history of LBP. However, its reliability for the assessment of thoracolumbar JPS remains questionable. The double inclinometer method is able to provide similar recordings with the tape measure method for the assessment of thoracolumbar ROM, but its recordings for the thoracolumbar JPS are quite different. The double inclinometer method seems to be appropriate for the assessment of the thoracolumbar ROM, but its application for assessing thoracolumbar JPS should wait until new studies with more promising findings are performed.

## Figures and Tables

**Table 1:** Demographics of patients with low back pain (*n* = 20, m/f 8/12).

Parameter	Μ (SD)
Age (years)	22.45 (4.72)
Height (cm)	175.55 (12.33)
Weight (kg)	75.60 (15.53)
NPRSpain (/10)	5.10 (1.61)
ODI (/50)	6.60 (4.13)
Pain chronicity (weeks)	2.50 (2.48)

NPRS: Numeric Pain Rating Scale; ODI: Oswestry Disability Index.

**Table 2:** Intra-rater reliability of the double inclinometer method for the assessment of thoracolumbar joint position sense.

Trials	Grand Mean	ICC	95%CI	SEM	SDD
Trials 1–5 (°)	5.78	0.74	0.58–0.87	2.48	6.86
Trials 1–2 (°)	5.85	0.79	0.55–0.91	2.47	6.84
Trials 1–3 (°)	5.43	0.74	0.54–0.87	2.66	7.36
Trials 2–3 (°)	5.54	0.68	0.34–0.86	2.77	7.67
Trials 3–5 (°)	5.74	0.70	0.46–0.86	2.41	6.67
Trials 4–5 (°)	6.32	0.74	0.40–0.89	2.20	6.09

ICC: Intraclass Correlation Coefficient; 95%CI: 95% Confidence Intervals; SEM: Standard Error of Measurement; SDD: Smallest Detectable Difference.

**Table 3:** Inter-rater reliability of the double inclinometer method for the assessment of thoracolumbar joint position sense.

Trials	Grand Mean	ICC	95%CI	SEM	SDD
Mean trials 1–2 (°)	5.56	0.59	0–0.83	3.35	9.27
Mean trials 1–3 (°)	5.63	0.62	0.03–0.85	3.05	8.44
Mean trials 1–4 (°)	5.92	0.61	0–0.84	3.09	8.55
Mean trials 1–5 (°)	5.91	0.62	0.03–0.85	2.96	8.19

ICC: Intraclass Correlation Coefficient; 95%CI: 95% Confidence Intervals; SEM: Standard Error of Measurement; SDD: Smallest Detectable Difference.

**Table 4:** Test-retest reliability of the double inclinometer method for the assessment of thoracolumbar joint position sense.

Trials	Grand Mean	ICC	95%CI	SEM	SDD
Mean trials 1–2 (°)	4.96	0.13	0–0.64	4.32	11.96
Mean trials 1–3 (°)	4.85	0.17	0–0.67	4.06	11.24
Mean trials 1–4 (°)	5.26	0.16	0–0.67	4.09	11.32
Mean trials 1–5 (°)	5.44	0.15	0–0.67	3.98	11.02

ICC: Intraclass Correlation Coefficient; 95%CI: 95% Confidence Intervals; SEM: Standard Error of Measurement; SDD: Smallest Detectable Difference.

**Table 5:** Intra-rater reliability of the double inclinometer method for the assessment of thoracolumbar range of movement.

Trials	Grand Mean	ICC	95%CI	SEM	SDD
Trials 1–2 (°)	63.60	0.88	0.72–0.95	6.20	17.17
Trials 2–3 (°)	65.81	0.94	0.86–0.97	4.25	11.77
Trials 1–3 (°)	64.59	0.88	0.77–0.94	5.91	16.37

ICC: Intraclass Correlation Coefficient; 95%CI: 95% Confidence Intervals; SEM: Standard Error of Measurement; SDD: Smallest Detectable Difference.

**Table 6:** Inter-rater reliability of the double inclinometer method for the assessment of thoracolumbar range of movement.

Trials	Grand Mean	ICC	95%CI	SEM	SDD
Mean Trials 1–2 (°)	63.21	0.90	0.76–0.96	7.74	21.43
Mean Trials 2–3 (°)	64.36	0.91	0.78–0.96	7.48	20.71
Mean Trials 1–3 (°)	63.61	0.91	0.79–0.96	7.26	20.11

ICC: Intraclass Correlation Coefficient; 95%CI: 95% Confidence Intervals; SEM: Standard Error of Measurement; SDD: Smallest Detectable Difference.

**Table 7:** Test-retest reliability of the double inclinometer method for the assessment of thoracolumbar range of movement.

Trials	Grand Mean	ICC	95%CI	SEM	SDD
Mean Trials 1–2 (°)	63.91	0.93	0.82–0.97	6.46	17.89
Mean Trials 2–3 (°)	66.15	0.91	0.79–0.96	6.87	19.02
Mean Trials 1–3 (°)	64.99	0.93	0.83–0.97	6.03	16.70

ICC: Intraclass Correlation Coefficient; 95%CI: 95% Confidence Intervals; SEM: Standard Error of Measurement; SDD: Smallest Detectable Difference.

## Data Availability

Data is unavailable due to privacy and ethical restrictions.
